# Deep Learning Based Greenhouse Image Segmentation and Shoot Phenotyping (DeepShoot)

**DOI:** 10.3389/fpls.2022.906410

**Published:** 2022-07-13

**Authors:** Narendra Narisetti, Michael Henke, Kerstin Neumann, Frieder Stolzenburg, Thomas Altmann, Evgeny Gladilin

**Affiliations:** ^1^Molecular Genetics, Leibniz Institute for Plant Genetics and Crops (IPK), Seeland, Germany; ^2^Automation and Computer Sciences Department, Harz University of Applied Sciences, Wernigerode, Germany

**Keywords:** greenhouse image analysis, image segmentation, deep learning, U-net, quantitative plant phenotyping

## Abstract

**Background:**

Automated analysis of large image data is highly demanded in high-throughput plant phenotyping. Due to large variability in optical plant appearance and experimental setups, advanced machine and deep learning techniques are required for automated detection and segmentation of plant structures in complex optical scenes.

**Methods:**

Here, we present a GUI-based software tool (DeepShoot) for efficient, fully automated segmentation and quantitative analysis of greenhouse-grown shoots which is based on pre-trained U-net deep learning models of arabidopsis, maize, and wheat plant appearance in different rotational side- and top-views.

**Results:**

Our experimental results show that the developed algorithmic framework performs automated segmentation of side- and top-view images of different shoots acquired at different developmental stages using different phenotyping facilities with an average accuracy of more than 90% and outperforms shallow as well as conventional and encoder backbone networks in cross-validation tests with respect to both precision and performance time.

**Conclusion:**

The DeepShoot tool presented in this study provides an efficient solution for automated segmentation and phenotypic characterization of greenhouse-grown plant shoots suitable also for end-users without advanced IT skills. Primarily trained on images of three selected plants, this tool can be applied to images of other plant species exhibiting similar optical properties.

## 1. Introduction

Image-based high-throughput plant phenotyping became a method of choice in quantitative plant sciences aiming to reveal casual links between phenotypic and genomic plant traits under varying environmental conditions (Li et al., 2014). The ultimate goal is to make an assessment of plant phenotypic traits data as efficient and scalable as genomic screening (Miller et al., 2007; Fahlgren et al., 2015). However, efficient and accurate processing and analysis of large image data from different optical set-ups represent a challenging task constituting one of the major bottlenecks in the pipeline of phenome-genome correlation (Minervini et al., 2015).

The first critical step in quantitative analysis of plant image data is image segmentation, which aims to classify all image pixels into two or more distinctive classes, e.g., foreground (plant) and background (non-plant) regions. Due to several natural and technical factors, segmentation of plant structures from background regions renders a challenging task. Inhomogeneous illumination, shadows, occlusions, reflections, and dynamic optical appearance of growing plants complicate the definition of invariant criteria for detection of different parts (e.g., leaves, flowers, fruits, and spikes) of different plant types (e.g., arabidopsis, maize, and wheat) at different developmental stages (e.g., juvenile, adult) in different views (e.g., top or multiple side views) (Henke et al., 2019). Consequently, conventional methods that are typically based on the some suitable image features and tailored to a particular data cannot be extended to new data in a straight forward manner. For example, one such popular approach to unsupervised image segmentation is based on analysis of differences between plant-containing and ‘empty' reference images (Choudhury et al., 2018). Thereby, it is assumed that background intensity/colors remain unchanged after plants were moved into the photo chamber. However, due to shadows and reflections both background and plant regions change their optical appearance. Moreover, these changes are dynamically progressing in course of plant development. Consequently, an 'empty' background image does not provide an ideal reference for the straightforward segmentation of plant structures. Mapping of RGB images onto alternative color spaces such as HSV and/or L*a*b is known to be useful for the separability of fore and background colors (Philipp and Rath, 2002; Pape and Klukas, 2015; Henke et al., 2021). However, it cannot completely solve the problem of overlapping plant and background colors.

To overcome the above limitations of uni-modal image analysis, a registration-classification approach to plant image segmentation was suggested in our previous study (Henke et al., 2020), which relies on pre-segmentation of plant regions in image modalities with higher fore-/background contrast, such as fluorescence images, followed by their co-registration with low-contrast image modalities (e.g., visible light or near-infrared images). Since segmentation masks derived from one image modality do not perfectly match another image modality, classification of plants and marginal background structures in masked image regions has to be subsequently performed using pre-trained intensity/color models. In some rare cases of severe plant movements due to the relocation of carriers from one photo chamber to another one, substantial differences between plant contours in two different image modalities can occur. Although the registration-classification showed relatively high accuracy of final segmentation results, the principle requirement of high-contrast multimodal data and occasional movement artifacts limit its application to experiments where only one single image modality (typically visible light images) is acquired.

Numerous further supervised approaches to intensity-/color-based plant image segmentation were proposed in the past. In Lee et al. (2018), automated segmentation of arabidopsis top-view images using a super pixel- and random forest classification-based algorithm was presented. In this approach, pre-labeled masks were used to segment each plant from the multi-tray experiment. However, like many other color-based models it is limited to a particular experimental setup and plant type. More recently, Adams et al. (2020) proposed a neural network based shallow learning method for the segmentation of side view visible light images. This approach classifies each pixel based on neighborhood pixel information of the trained ground truth data and outperforms conventional thresholding methods.

All the above state-of-the-art techniques require reference images, the presence of particular image features, and expertise in manual parameter tuning for each image to be segmented. Consequently, conventional supervised techniques are typically trained on and applied to particular types of plants, experimental set-ups, and illumination scenes. However, high-throughput phenotyping of thousands and millions of plant images demands fully automated, efficient, and accurate segmentation algorithms with higher order cognitive abilities that can tolerate variation in scene illumination and plant/background colors.

In recent times, convolutional neural networks (CNNs) gained high attention especially in computer vision applications, because of the ability to directly extract and train relevant multi-level features from data without prior knowledge and human effort in feature design. CNNs have been shown to outperform conventional approaches when applied to many traditionally difficult tasks of image analysis including pattern detection and object segmentation in biomedical images (Ronneberger et al., 2015; Bai et al., 2018), traffic scenes (Badrinarayanan et al., 2017) and remote sensing (Marmanis et al., 2016). In recent years, they were also used for high-throughput plant phenotyping such as the detection of wheat roots grown in germination paper (Pound et al., 2017), segmentation of roots from the soil in X-ray tomography (Douarre et al., 2018), and segmentation of spikes in wheat plants (Misra et al., 2020). However, most of these studies present exemplary applications and/or computational frameworks that can hardly be handled by end-users without advanced programming skills.

The aim of this study is to develop an efficient and handy tool for automated shoot image segmentation and quantification for different plant types using a pre-trained deep CNN framework which could be used in a straight forward manner even by unskilled users. The GUI software tool (**DeepShot**) developed for this purpose relies on the U-net segmentation model from Ronneberger et al. (2015) which was trained on ground truth images of three different plants (arabidopsis, maize, and barley) acquired from two different views (side, top) in different stages of their development. The article is structured as follows. First, we present our methodological framework including the proposed U-net based framework for shoot image segmentation, ground truth data generation, and training and evaluation procedures. Then, the results of experimental investigations are presented including a model performance by application to segmentation of test shoot images vs. alternative state-of-the-art solutions.

## 2. Materials and Methods

### 2.1. Image Data

**Figure 1:**
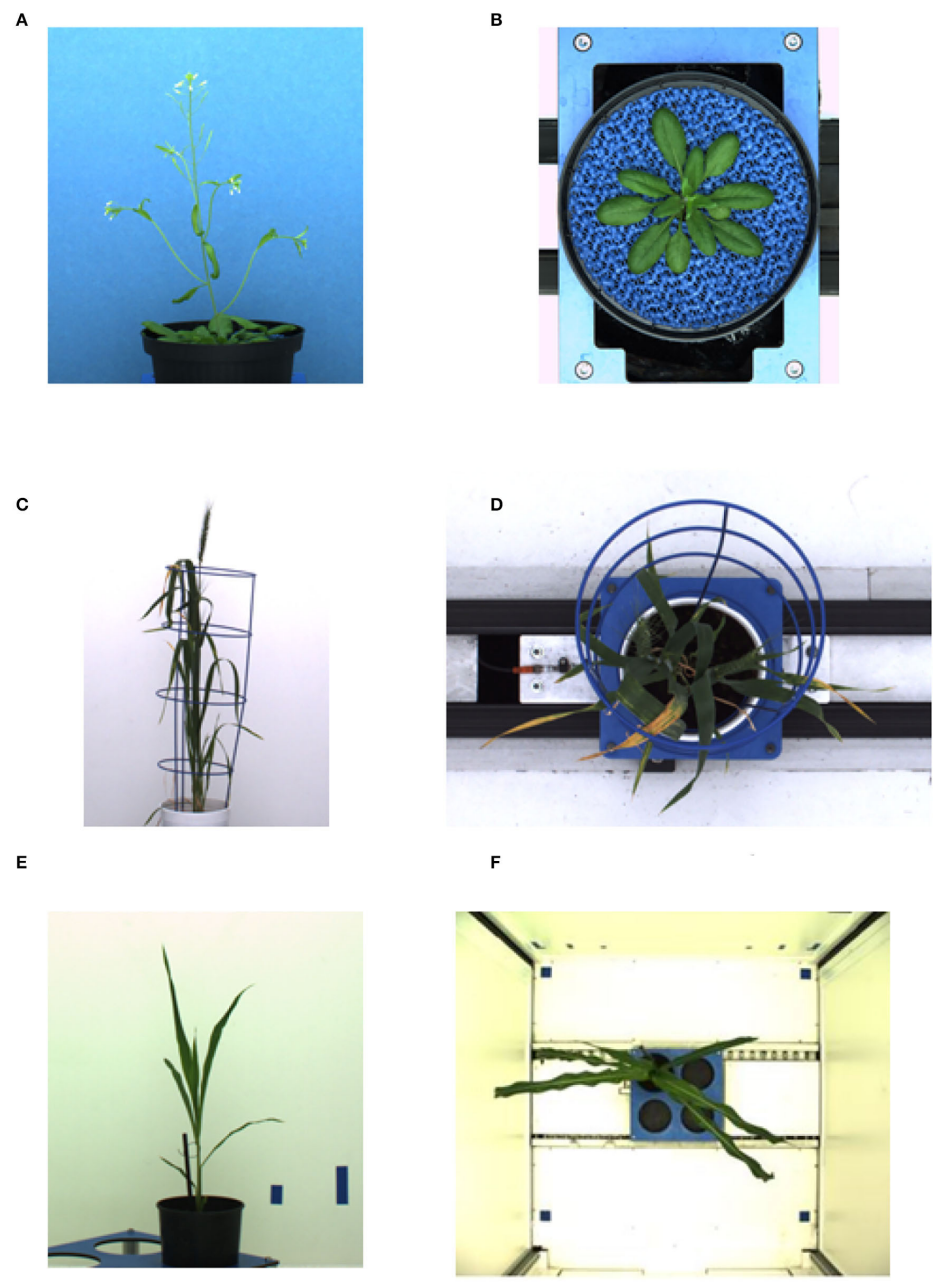
Examples of side- and top-view images of arabidopsis **(A,B)**, barley **(C,D)**, and maize **(E,F)** plants acquired with three different plant phenotyping platforms.

The deep learning-based shoot image analysis tool (DeepShoot) is designed for automated segmentation and quantification of visible light (VIS) images of arabidopsis, maize, and barley shoots acquired from greenhouse phenotyping experiments using LemnaTec-Scanalyzer3D high throughput plant phenotyping platforms (LemnaTec GmbH, Aachen, Germany). Figure 1 shows examples of arabidopsis, maize, and barely images from three different LemnaTec phenotyping platforms tailored to the screening of large, mid-size, and small plants. All three phenotypic platforms have different designs of photo chambers, illumination, colors of background walls, and camera resolutions ranging between 1 and 6 Mpx.

### 2.2. Ground Truth Generation

For the training of CNN segmentation models, a representative set of ground truth images with an accurate annotation of fore- and background image regions is required. In this study, the generation of ground truth images of different greenhouse cultured plants was performed using the GUI software tool kmSeg (Henke et al., 2021), which allows for the efficient annotation of image regions by manual selection of pre-calculated k-means color clusters corresponding to targeted plant structures. Background structures that exhibit similar color fingerprints as plant regions and, thus, could not be separated by color clustering are excluded or subsequently removed using manual region masking and cleaning likewise provided with the kmSeg tool. Semi-automated segmentation of a typical greenhouse image using kmSeg takes between 1–5 min depending on color composition and structural complexity of a given plant shoot image.

### 2.3. Image Segmentation Using CNN

The proposed CNN model is derived from the original encoder-decoder architecture of U-net (Ronneberger et al., 2015), which provides a versatile framework for semantic segmentation. In our model, batch normalization (Ioffe and Szegedy, 2015) is applied after each convolution layer in contrast to the original U-net. Because batch normalization improves the network performance and stability by normalizing the feature maps at respective levels (Ioffe and Szegedy, 2015; Santurkar et al., 2019). Furthermore, the original U-net used dropout layers to remove outliers in the feature maps. But we avoided this layer because the combination of batch normalization and dropout layers can cause worse results (Li et al., 2018). Also, to improve the segmentation quality on largely connected patterns, a larger kernel size is considered in our approach compared to the original U-net (Peng et al., 2017). Finally, our CNN model has less depth (of 3) compared to the original U-net depth of 4 due to the smaller input image size. The detailed comparison of convolutional parameters with respect to the original U-net is summarized in Table 1.

**Table 1:** Convolutional parameters of the original U-net and proposed modifications.

**Convolutional parameters**	**Original U-net**	**Proposed modifications**
Kernel size	3 x 3	7 x 7
Transposed kernel size	2x2	3x3
Stride	1 x 1	2 x 2
Padding	Unpadded	Padding with zeros
Depth	4	3
Number of filters	(64, 128, 256, 512, 1,028)	(16, 32, 64, 128)

**Figure 2:**
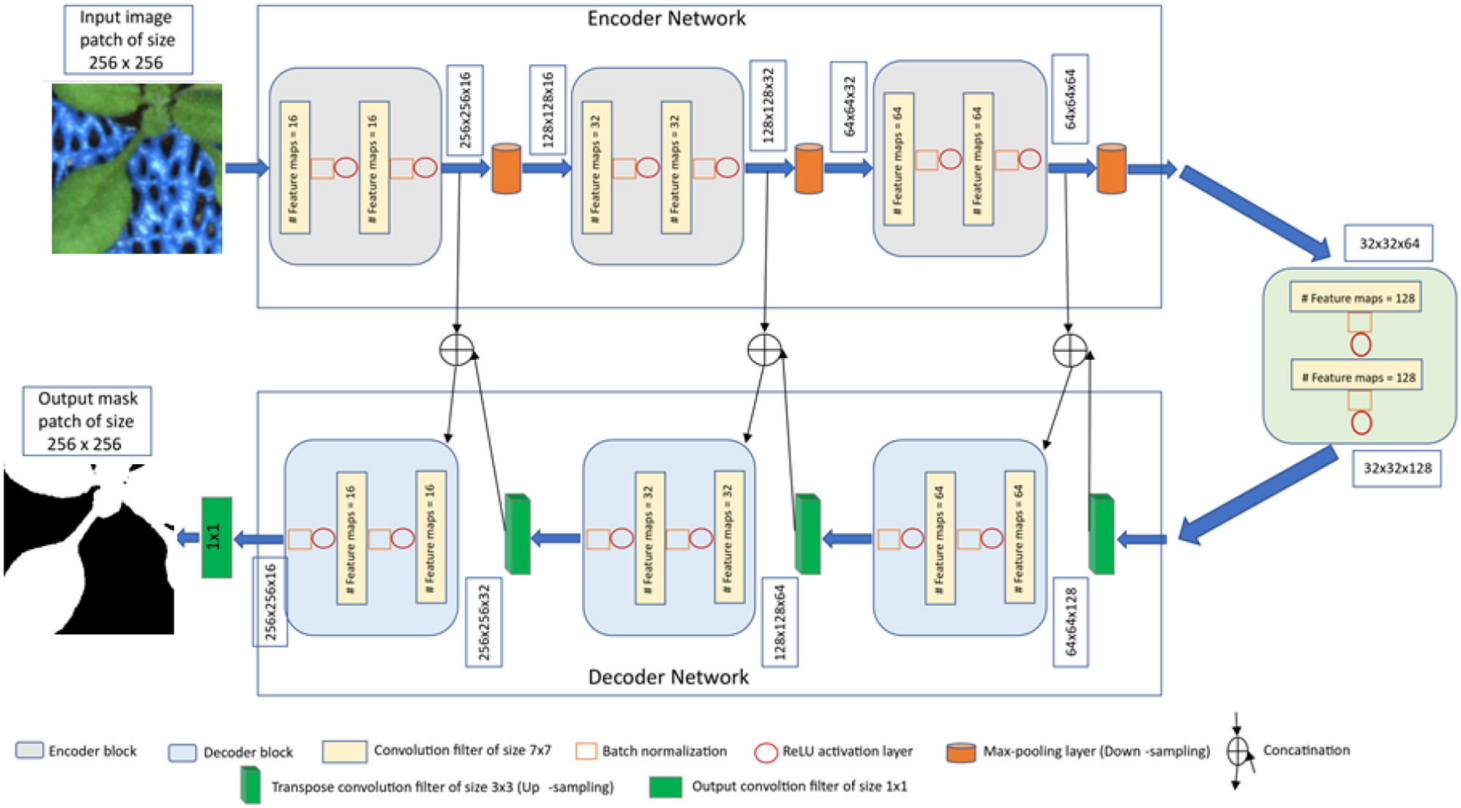
The proposed U-net architecture for shoot image segmentation.

Under consideration of the above suggested modifications, the U-net framework was adapted to the task of multimodal shoot image segmentation, refer to Figure 2. This network is designed in such a way that training and testing are performed on patches of input images in original resolution. The advantage of this image masking approach is that it enables model training using a large amount of ground truth data on consumer GPUs without losing high frequency information due to image downscaling. Furthermore, training of CNNs on image patches is more advantageous for learning local features than full-size images (Jha et al., 2020). Therefore, the input and output layers of the network are designed to operate on images of the size 256 x 256. Further details of the network encoder and decoder layers are described below.

**Encoder network**: The encoder network consists of 3 encoder blocks. The first encoder block takes the image patches of size 256 x 256 as input and produces corresponding feature maps of size (256 x 256 x 16) as output. Then, the feature maps are forwarded to the second and third encoder blocks to generate further feature maps for the plant pixel detection. Each encoder block consists of two convolutional layers to learn feature maps at respective levels, where each convolutional layer consists of a 7 × 7 convolution filter followed by batch normalization (Ioffe and Szegedy, 2015) and a non-linear activation function called Rectified Linear Unit (ReLU) (Agostinelli et al., 2014). Followed by each encoder block, a max-pooling operation using a general window size of 2 x 2 (Wang et al., 2015; Jha et al., 2020) is applied for down-sampling the feature maps by half of their original size. The above steps enable a more efficient aggregation of image features. All three encoders are repeated with varying depths of 16, 32, and 64 to detect diverse plant features respectively.

Followed by the encoder network, a bridge encoder block without a max-pooling layer is applied. This results in 128 feature maps of the size 32 x 32.

**Decoder network**: The output from the bridge encoder (32 x 32 x 128) is upsampled using 3 x 3 transpose convolution with the same padding and stride 2. This means the size of feature maps (32 x 32 x 128) was doubled to (64 x 64 x 128) by applying the filter of size 3 x 3 to all input elements and boarder elements were computed using zero padding. Then the resulting feature map is concatenated with the corresponding encoder feature maps. This results in feature maps of size (64 x 64 x 256) that are subsequently passed through a convolutional layer like an encoder block but have decreasing channel depth of 64. This process is repeated for the remaining decoder blocks with decreasing channel depths of 32 and 16. Finally, the output of the final decoder block is fed into a convolutional layer of size 1 x 1 x 1 with a “Softmax” activation function (Dunne and Campbell, 1997) to classify each pixel as plant or non-plant at the patch level. The output of the proposed architecture is a predicted mask of size 256 x 256 like the input image patch shown in Figure 2.

### 2.4. Performance Metrics

To evaluate the performance of the proposed U-net model during the training and testing stage, the Dice coefficient (DC) (Zou et al., 2004) is used. It measures the area of intersection between the model and ground truth segmentation and its value ranges from 0 to 1, where 1 corresponds to 100% perfect and 0 to false segmentation. The Dice coefficient is defined as:


(1) $$\begin{array}{r} \begin{array}{c} DC=\frac{2*(P\cap G)}{P\cup G}=\frac{2*\sum_{i}^{N}P_{i}G_{i}}{\sum_{i}^{N}P_{i}+\sum_{i}^{N}G_{i}} \end{array} \end{array}$$

where P and G are predicted and ground truth binary images, respectively. *P*_*i*_ and *G*_*i*_ are output values 0 and 1 of pixel i in predicted and ground truth binary images, respectively.

### 2.5. Computational Implementation

#### 2.5.1. Training

The proposed U-net architecture was developed under Python 3.8 using TensorFlow (Abadi et al., 2016) with Keras API. In addition, image processing operations such as reading, cropping, and training data preparation were done using PIL, Numpy (Walt et al., 2011), and Scikit-Image (Van der Walt et al., 2014) packages. Then the proposed model was trained on a GPU machine with Linux operating system (Intel(R) Core (TM) i7-10700K CPU @ 3.80GHz) and NVIDIA RTX 3090-24GB graphic card. As discussed above, the model is designed in such a way that training will be performed on patches of the original image. Thus, to generate non-overlapping patches of size 256 x 256, original images were padded with zeros at the image edges so that their width and height are divisible by 256. Out of these non-overlapping patches, both plant and background masks are considered in equal proportion to avoid potential imbalance between plant and non-plant training masks. Then each cropped mask is normalized in the range of [0, 1] for feature consistency in the CNN network. The overview of prepared training data of arabidopsis, barley, and maize and their growth stages are described in Tables 2, 3, respectively. Regarding information on growth stages, an approximately equal number of images from different developmental stages (early, mid, and late developmental phases) were analyzed in this study.

**Table 2:** Overview of training data including images of arabidopsis, barley, and maize plants.

		**Side view**		**Top view**	
**Plant**	**Original resolution**	**# of images**	**# of masks**	**# of images**	**# of masks**
Arabidopsis	2,056 x 2,454	197	17,730	193	17,730
Barley	1,234 x 1,624	100	3,395	100	1,908
Maize	2,056 x 2,454	100	3,669	55	1,036

**Table 3:** Overview of growth stages of training data from arabidopsis, barley, and maize plants.

	**Growth stage**			
**Plant/View**	**# of images**	**Early**	**Mid**	**Late**
Arabidopsis/Side	197	62	65	70
Arabidopsis/Top	193	62	65	66
Barley/Side	100	30	35	35
Barley/Top	100	30	35	35
Maize/Side	100	30	35	35
Maize/Top	50	20	20	15

Subsequently, based on our experience and previous studies (Crimi et al., 2018; Joseph, 2022), the above prepared data sets were partitioned into training and validation in the ratio of 85:15, respectively. The initial weights of the proposed model were defined randomly with zero mean and SD of 0.05 as proposed by Krizhevsky et al. (2012). Then the model was optimized with an Adam optimizer (Kingma and Ba, 2014) to improve the segmentation performance on training data sets. The binary cross-entropy loss function (Jha et al., 2020) is used to measure the unhappiness of the model during training and it defines the difference between predicted output and ground truth generated by the kmSeg tool as described above. This function compares each pixel prediction (0: non-plant, 1: plant) with the ground truth pixel and averages all pixels loss for computing the total loss of the image. Therefore, each pixel contributes to the overall objective loss function. Then the model was trained for 100 epochs with 16 convolutional channel features and a batch size of 128 as per system constraints. The learning rate alters the magnitude of the updates to the model weights during each iteration and is initialized with 0.001. Then a learning rate scheduler was used to dynamically reduce the learning rate by a factor of 0.2 if the validation loss is not improved in the next 5 iterations. This was introduced in order to avoid a too quick convergence of the model to a suboptimal solution and overfitting in the case of a large learning rate. Whereas, a too small learning rate may never converge and get stuck on the suboptimal solution (Bengio, 2012). Here, note that all data sets (arabidopsis, barley, and maize) were trained in a similar way with the same parameter configuration.

#### 2.5.2. Prediction

**Figure 3:**
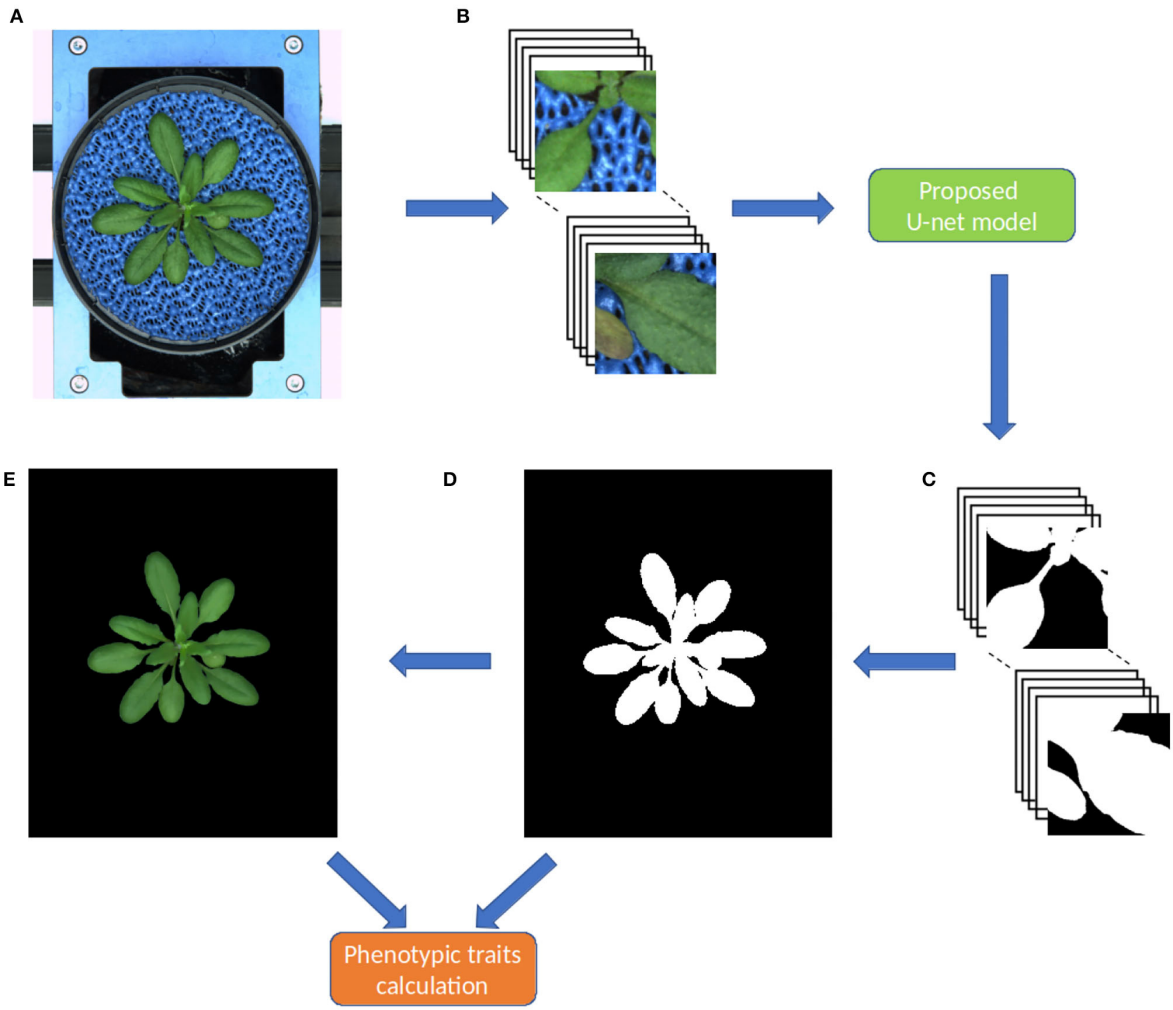
Workflow of the pipeline for image processing and segmentation in the DeepShoot tool. Green and orange color boxes represent the operations of image segmentation and trait calculation: **(A)** original image, **(B)** original image patches of size 256 x 256, **(C)** segmented image patches of size 256 x 256, **(D)** binary segmentation of the original image, **(E)** RGB color space of **(D)**.

As stated above, original shoot images have a variational resolution, whereas the proposed model requires input images of the size 256 x 256. Thus, during the prediction stage, original images are padded with zeros then non-overlapping 256 x 256 masks were generated similar to what was done in the training stage. The model does predictions on these 256 x 256 masks then they are combined into a single output image as shown in Figure 3. This process is dynamic, that means any image with a resolution greater which 256x256 can be segmented in an automated manner.

Since the output layer of the model is a Sigmoid activation function or logistic function, the predicted segmentation is a probability map with values ranging in between 0 and 1. Hence, this probability map is converted to a binary image using threshold 'T'. Here, relatively, plant pixels will have high probabilities compared to the background pixels. Therefore, T ≥ 0.6 is chosen to consider all high probability pixels as plant pixels in the final segmentation. After fully automated segmentation, phenotypic traits of segmented plant structures were calculated in the final step.

#### 2.5.3. Graphical User Interface

In practice, end-users prefer to have an easy-to-use software solution with a Graphical User Interface (GUI). Therefore, a user-friendly GUI front-end was developed under the MATLAB 2021a environment (MATLAB Optimization Toolbox, 2021) to comfortably operate the complex algorithmic framework of shoot segmentation software. Figure 3 shows the complete workflow involved in the DeepShoot tool for automated plant segmentation and trait extraction. For import of deep learning models trained under Python the MATLAB interoperability routine *importKerasNetwork* (MATLAB Optimization Toolbox, 2021) was used. According to the specification of this function, the U-net models trained in Python were exported in the so-called h5 file format, which is supported by the recent versions of MATLAB including 2021a.

### 2.6. Method Comparison

The performance of our proposed model is compared with the recently published shallow learning based neural network (NN) by Adams et al. (2020) which was developed and evaluated for the same application as ours, namely, segmentation of greenhouse shoot images. This algorithm classifies each pixel based on 3x3 neighborhood information from red, green, and blue channels using fully-connected neural networks. In this study, the same NN model architecture was retrained on our image data set with a large number of neighborhood features of 5,939,562 and a higher batch size of 4,096 compared to the original study of 51,353 and 1,024, respectively.

In addition, the proposed encoder backbone of the U-net architecture is compared with different encoder backbones including vgg19 (Simonyan and Zisserman, 2014), resnet50 (He et al., 2016), and xception (Chollet, 2017). These models were trained on the same image data set with a similar training configuration except for the increased number of filters (64, 128, 256, 512) as discussed in Section 2.5.1.

## 3. Results

### 3.1. Training and Validation

**Figure 4:**
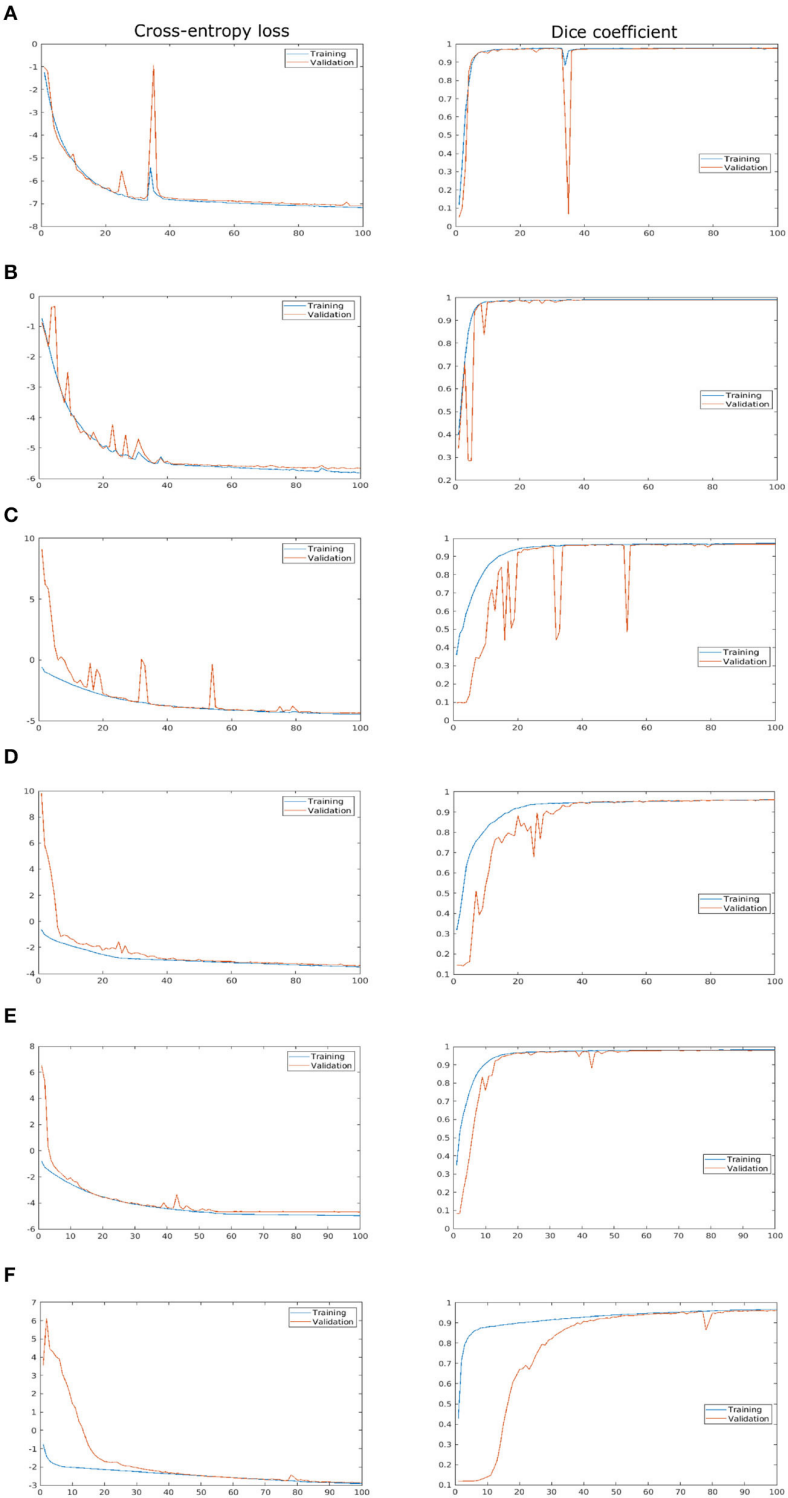
Training and validation performance of the shoot model over 100 epochs. X- and Y-axes represent the epoch number and performance measure, respectively. For visualization purposes, logarithmic cross-entropy values are plotted for all models. **(A)** Arabidopsis side view, **(B)** Arabidopsis top view, **(C)** Barley side view, **(D)** Barley top view, **(E)** Maize side view, and **(F)** Maize top view.

As described above, the proposed network was trained and validated on six different data sets including arabidopsis, barley, and maize images acquired from three different plant phenotyping facilities. Thereby, each of these three data sets was subdivided into training and validation sets in the ratio of 85:15, respectively. The model performance is analyzed using binary cross-entropy loss (CE loss) and Dice coefficient at each epoch during the learning stage of the network. Because of the dynamic optical appearance of growing plants, segmentation of shoot regions in side view images represents a more difficult task. This results in discontinuous shoot structures in segmented images. Therefore, it is important to give equal weights to errors related to both background and plant pixels in this study using the Dice coefficient. Figure 4 shows the training and validation performance of the proposed model on six different data sets over 100 epochs. It shows that the training loss of six models was minimized and platen the curve after epoch number 60. Simultaneously, training DC was maximized and achieved more than 90% of the accuracy for all models by the end of the training epochs. In turn, the generalized performance of the model is measured using validation measurements. Similarly, training performance and validation DC also achieved more than 90% accuracy with a low value of loss for all models at the end of the epochs. A brief overview of training and validation measurements is shown in Table 4.

**Table 4:** Training and validation performance of the shoot models.

	**Training**		**Validation**	
**Plant**	**CE loss**	**DC**	**CE loss**	**DC**
Arabidopsis side view	0.00075	0.9821	0.00083	0.9707
Arabidopsis top view	0.00297	0.9907	0.00345	0.9846
Barley side view	0.01172	0.9737	0.01300	0.9589
Barley top view	0.02986	0.9593	0.03366	0.9551
Maize side view	0.00687	0.9834	0.00906	0.9731
Maize top view	0.05433	0.9742	0.05673	0.9671

**Figure 5:**
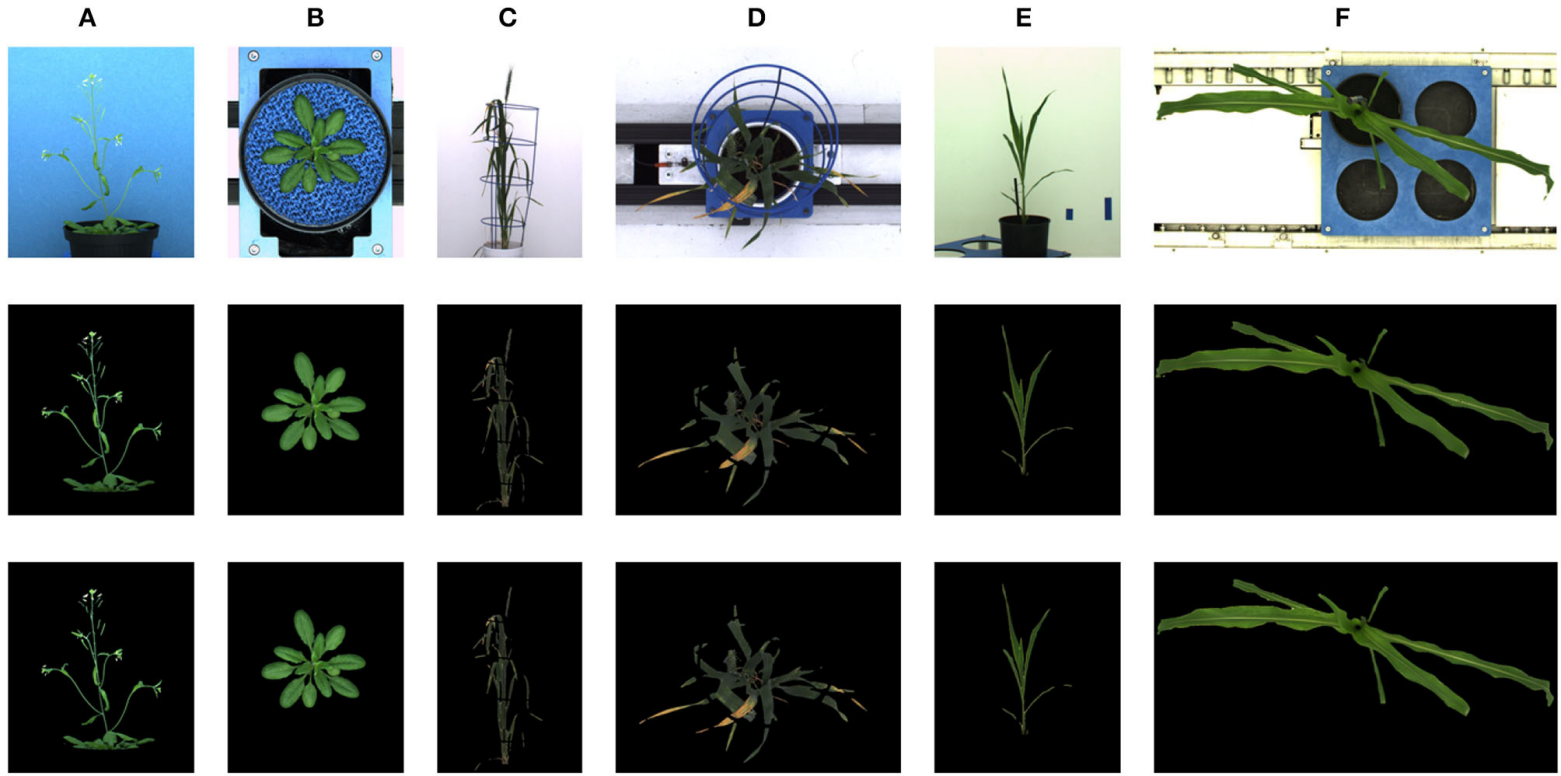
Segmentation performance: first, second and third row represents the original RGB image, ground truth segmentation by the kmSeg tool and predicted segmentation by the DeepShoot tool, respectively. The DC of each image as following: **(A)** Arabidopsis side view: 0.9117, **(B)** Arabidopsis top view: 0.9876, **(C)** Barley side view: 0.9384, **(D)** Barley top view: 0.9617, **(E)** Maize side view: 0.9709, **(F)** Maize top view: 0.9843.

**Figure 6:**
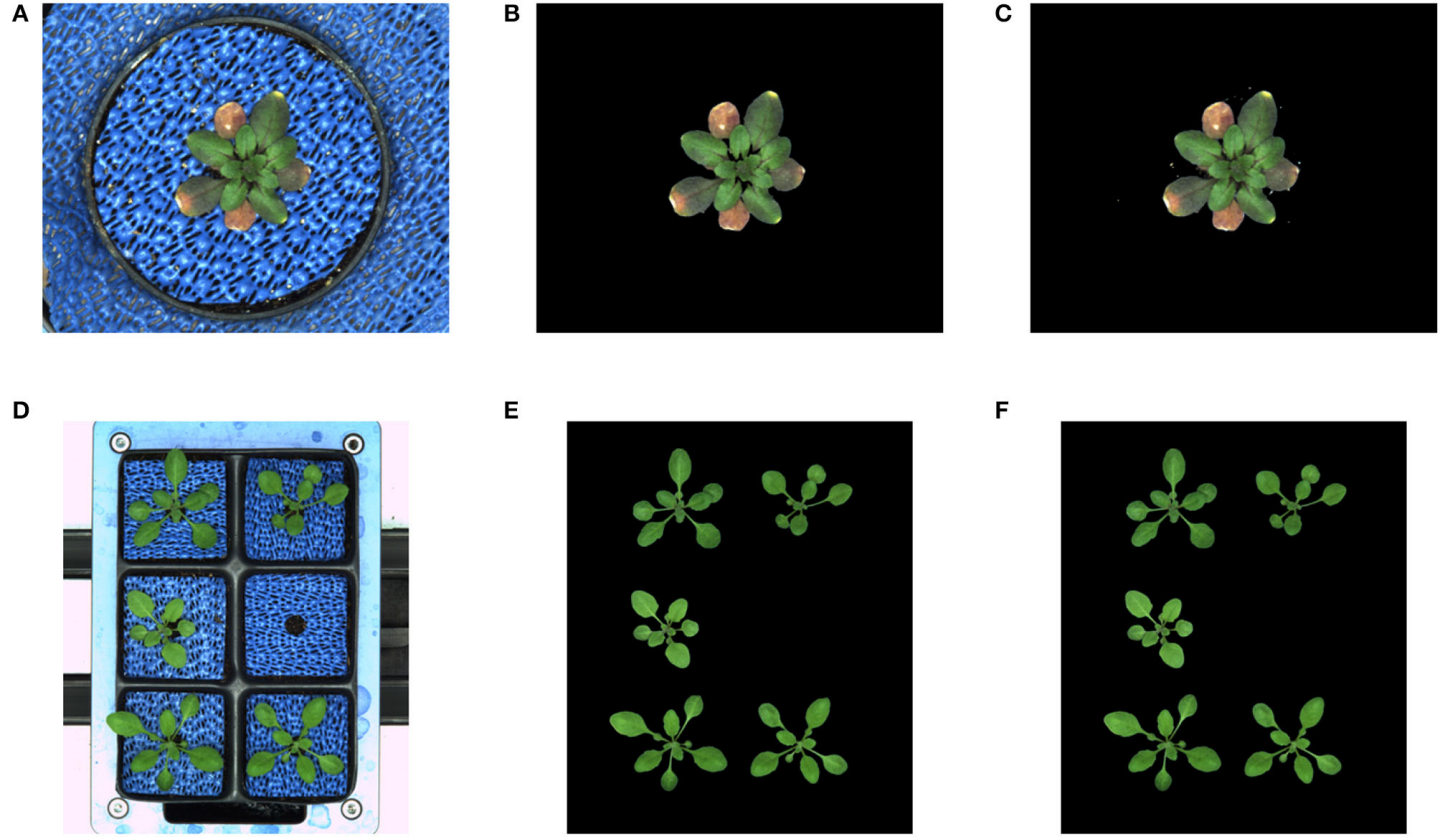
Segmentation performance on variational data sets: DC of stress and multi-tray experiments are 0.9664 and 0.9873, respectively. **(A)** Stress experiment image, **(B)** Ground truth, **(C)** Predicted segmentation, **(D)** Multi-tray image, **(E)** Ground truth, and **(F)** Predicted segmentation.

In addition to the training performance, the exemplary segmentation of all models on test images is shown in Figure 5. It turns out that all models performed with a relatively higher DC of 0.95 except for the arabidopsis side-view model which has a DC of 0.9117 compared to the ground truth generated by the kmSeg tool. Furthermore, trained models were tested on variational data sets from arabidopsis top-view like stress and multi-tray experiments as shown in Figure 6. Here, the model resulted in DC of 0.9664 and 0.9873 for stress and multi-tray experiments image compared to the ground truth, respectively.

### 3.2. Evaluation of the Reference Data Set

**Figure 7:**
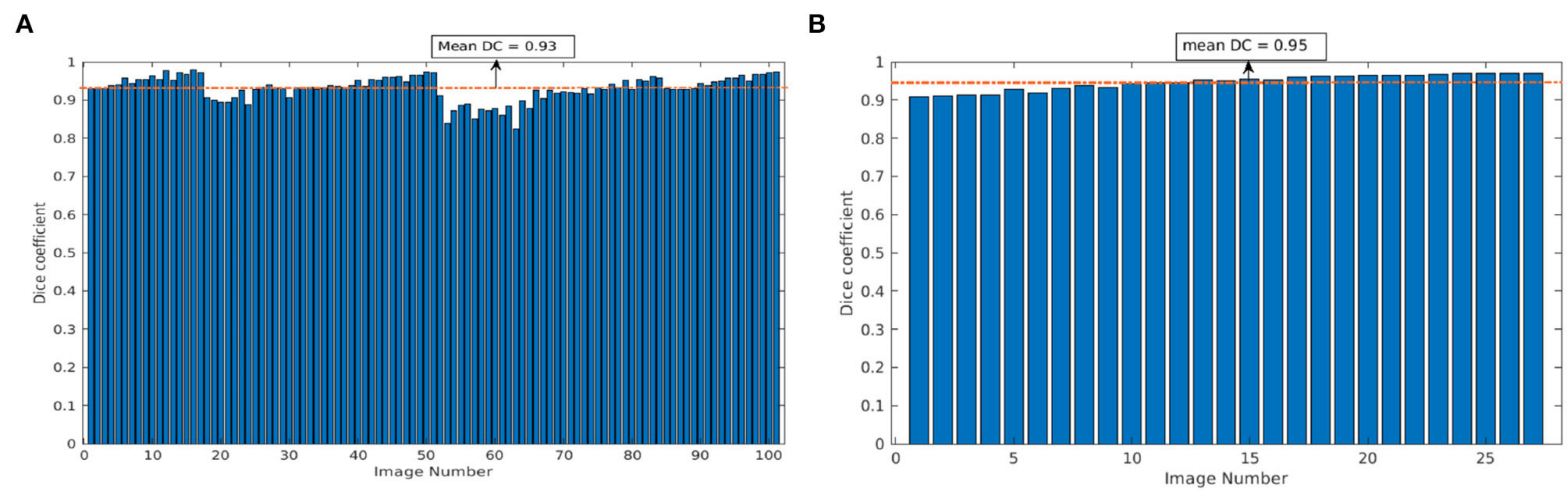
Evaluation of image segmentation on the references data set from Scharr et al.: Dice coefficient of arabidopsis top-view model over 100 and 27 images for single- and multi-tray experiments, respectively **(A,B)**. The dotted orange line represents the mean DC value.

**Figure 8:**
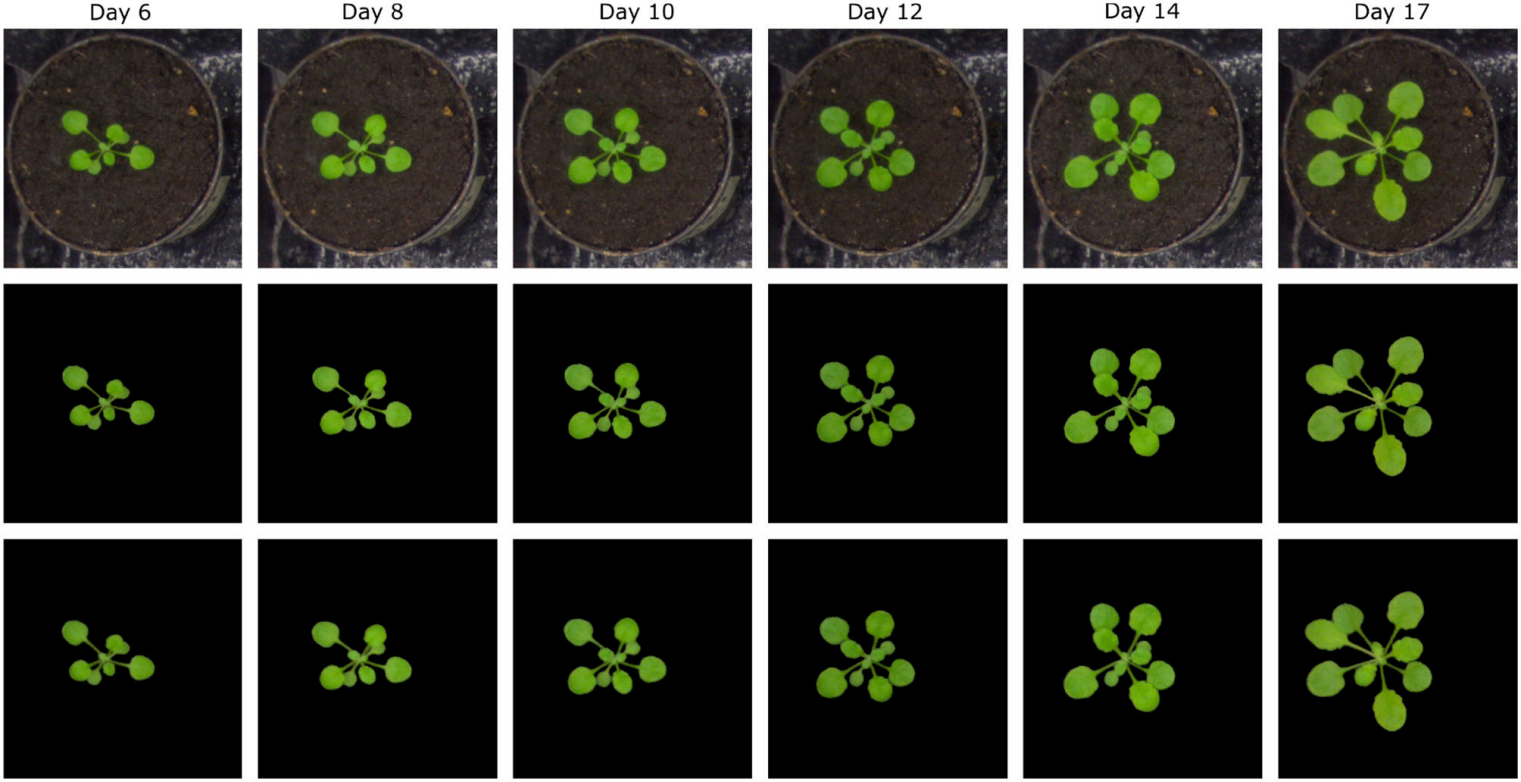
Examples of segmentation of arabidopsis top-view images from Scharr et al. All images were segmented with DC over 0.9.

To measure the performance of the model on unseen data, our CNN model trained on arabidopsis top-view images from LemnaTec-Scanalyzer3D was applied to the set of arabidopsis top-view from Scharr et al. (2016). This dataset was frequently used for CNN model training and evaluation in several previous studies within the scope of CVPPP competitions (https://www.plant-phenotyping.org/CVPPP2018, https://www.plant-phenotyping.org/CVPPP2019 https://www.plant-phenotyping.org/CVPPP2020). However, here it is only used for cross-validation of our model trained on images from our phenotyping facility. Figure 7 shows the mean DC of single and multi-tray experiments from the Scharr et al. data set. The model resulted in the mean DC of 0.93 over 100 images and 0.95 over 27 images for single and multi-tray experiments, respectively. Examples of segmentation of single-tray images from the references data set are shown in Figure 8.

### 3.3. Evaluation of DeepShoot vs. Alternative Solutions

**Figure 9:**
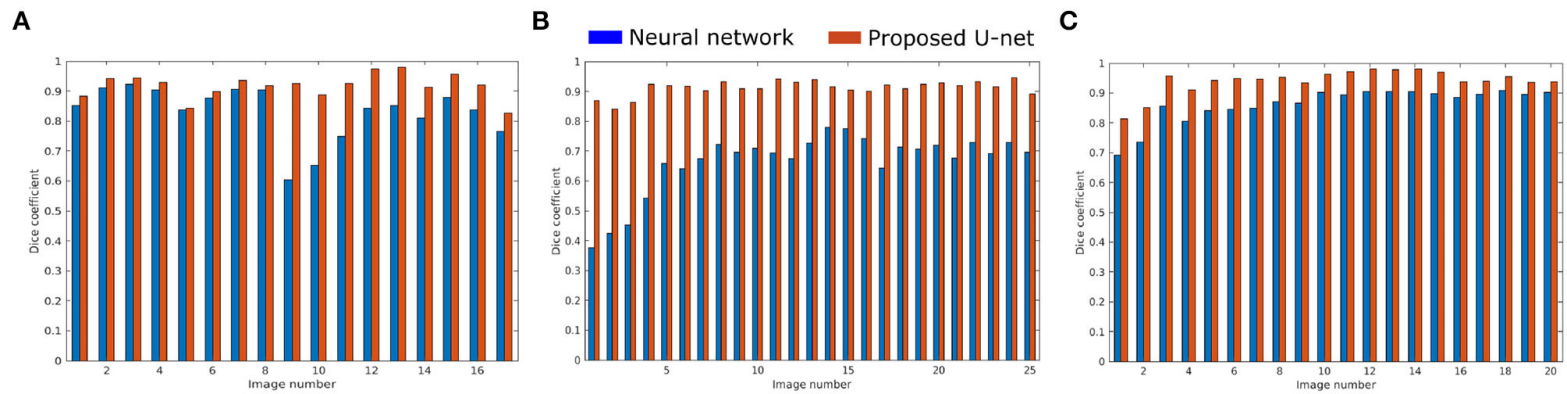
Performance of neural network (blue) and proposed U-net (orange) segmentation models on **(A)** 17 arabidopsis, **(B)** 25 barley, and **(C)** 20 maize side view images.

**Figure 10:**
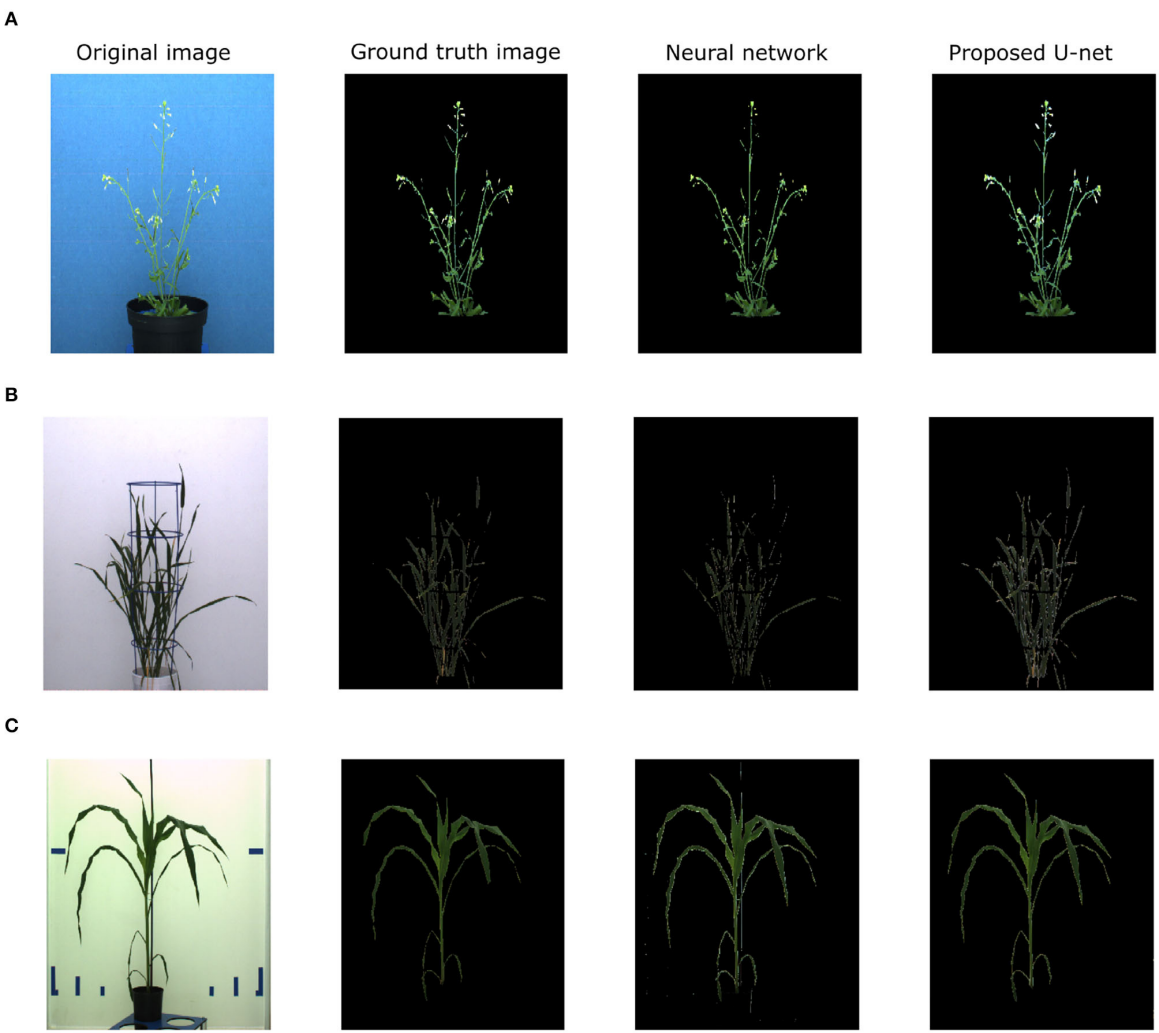
Evaluation of neural network segmentation with respect to the proposed U-net on arabidopsis, barley and maize side view images respectively. **(A)** NN DC: 0.7824, DeepShoot DC: 0.8342, **(B)** NN DC: 0.6973, DeepShoot DC: 0.8924, **(C)** NN DC: 0.8746, DeepShoot DC: 0.9360.

The proposed U-net was compared with the recently published shallow learning based neural network (NN) by Adams et al. (2020) which was originally developed and evaluated for shoot side view image segmentation. Figure 9 shows the comparative analysis of 17, 25, and 20 side view images of arabidopsis, barley, and maize plants, respectively. It briefs that the proposed U-net outperforms DC > 0.9 for all images, whereas neural networks predictions have DC between 0.5 and 0.8. An exemplary segmentation of three plants using a neural network and proposed U-net with respect to ground truth is shown in Figure 10. Also, the computational time of both segmentation models required for the prediction on Intel(R) Xeon(R) Gold CPU @2.10 GHz with 20 CPU cores is listed in Table 5.

**Table 5:** The computational time of shoot segmentation algorithms in seconds per image on a system with Intel(R) Xeon(R) Gold 6130 CPU @2.10GHz with 20 CPU cores.

**Algorithm**	**Arabidopsis (2,056 x 2,454)**	**Barley (1,234 x 1,624)**	**Maize (2,056 x 2,454)**
Neural networks	20,410	13,100	20,410
Proposed U-net	7.2	3.05	7.2

**Figure 11:**
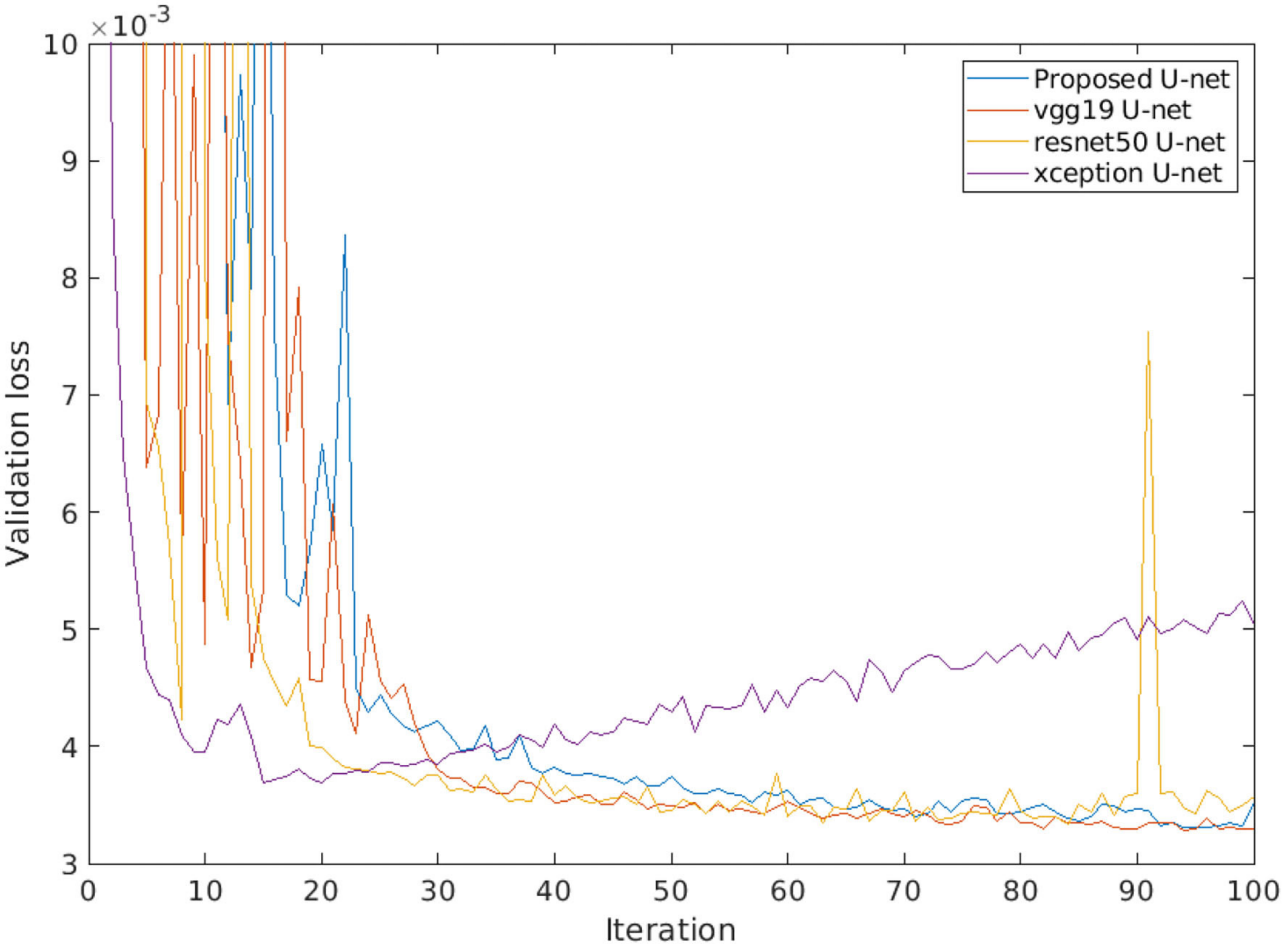
Loss performance of alternative U-net models with different encoder backbones on arabidopsis top view images.

Furthermore, a comparison of different encoder backbones (vgg19, resnet50, and xception) of the U-net architecture was performed. Figure 11 shows the performance of alternative U-net backbones by training on arabidopsis top view images. It shows that both resnet50 and xception networks have higher validation loss (>0.004) and it increases over several iterations. On the other hand, vgg19 and the proposed U-net are promising comparable performances with a lower validation loss of 0.0033. In addition, the complexity of alternative U-net models with different encoder backbones on arabidopsis top view images is shown in Table 6.

**Table 6:** The complexity of alternative U-net models with different encoder backbones on arabidopsis top view images.

**Parameter**	**Proposed U-net**	**vgg19**	**resnet50**	**xception**
# of trainable parameters	2,484,721	24,780,993	15,053,121	5,583,065

### 3.4. DeepShoot GUI Tool

**Figure 12:**
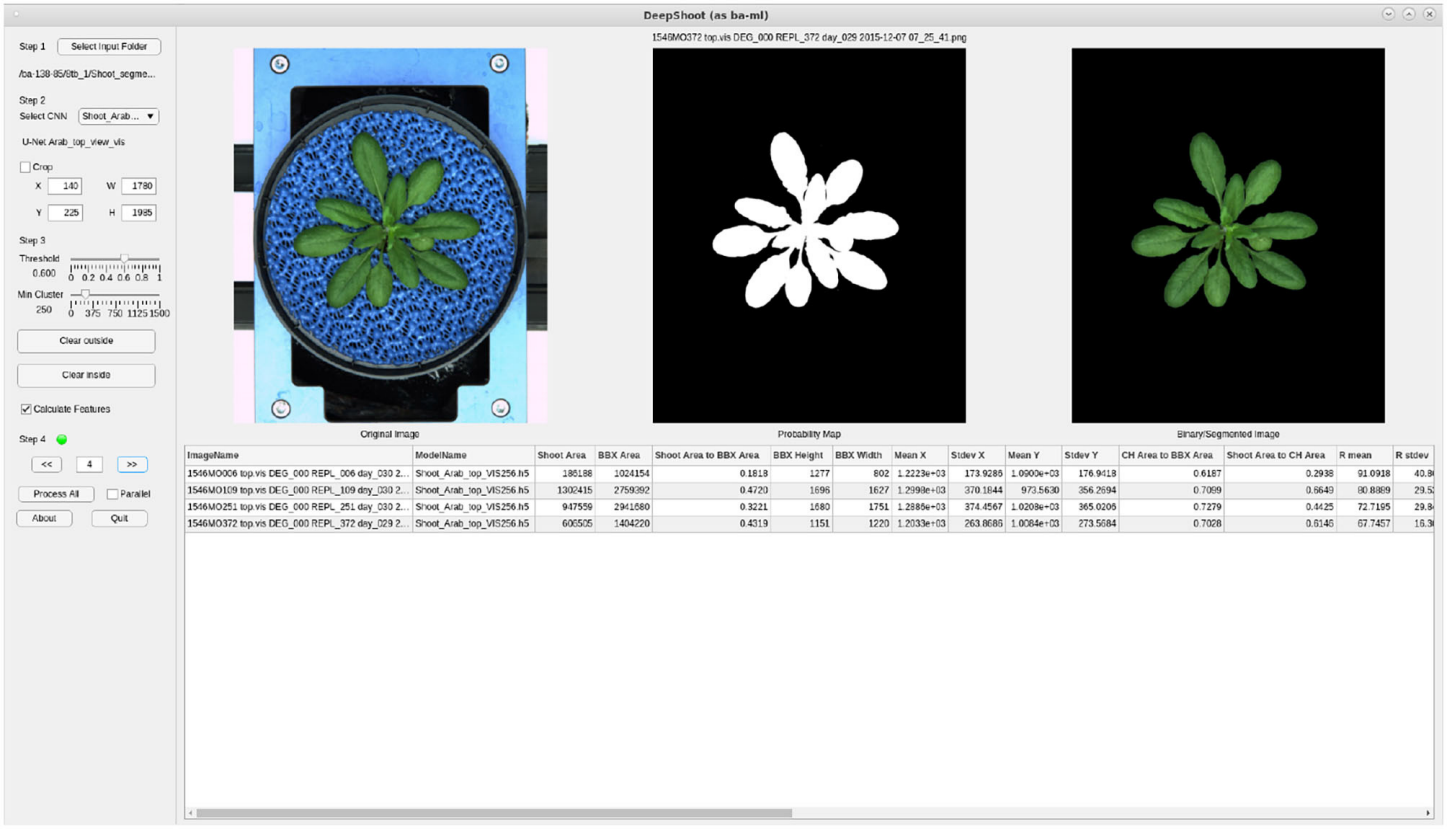
Graphical User Interface of the DeepShoot tool: left, middle, and right images represent the original image, predicted probability map, and predicted color image, respectively.

Figure 12 shows the GUI of DeepShoot software which is freely available as a precompiled executable program from https://ag-ba.ipk-gatersleben.de/ds.html. In addition to automated image segmentation, DeepShooot calculates 35 shoot traits that are categorized into 4 feature groups (i.e., area, bounding box traits, convex-hull area, and statistical color features). Further information on the definition of traits can be found in Supplementary Table 1 accompanying this article.

In order to restrict the analysis to the region of interest (ROI), users can define a custom ROI as a rectangle or polynomial shape using the crop or *Clear outside* buttons of the DeepShoot GUI. The DeepShoot tool can be applied for analysis of single images in a step-by-step manner or for automated processing of all images in a selected folder. Regarding DeepShoot time performance, image segmentation and traits calculation all together take an average of 18.5 s to process and analyze a 5-megapixel image on a system with Intel(R) Xeon(R) Gold 6130 CPU @2.10GHz with 20 CPU cores.

## 4. Discussion

Automated processing and quantitative analysis of a large amount of phenotypic image data represent a critical point in determining the efficiency and accuracy of trait computation. The deep learning-based tool for automated shoot image segmentation and phenotypic analysis developed in this study aims to address this challenging task. Our experimental tests on three different plant types (arabidopsis, barley, and maize) and two different views (side and top) showed that the performance of the model during the training is improved over the number of iterations. On the other hand, the model trained (for all plants) before iteration number 40 was under performed and showed worse performance for model validation. However, due to the dynamic reduction in learning rate by a factor of 0.2 a stable performance with more than 90% Dice coefficient for all shoot models was achieved. Additional information on the impact on learning rate can be found in Supplementary Figure 1 accompanied by this article. Moreover, arabidopsis and maize models achieved low CE loss values, whereas barley models have slightly higher CE loss values due to the variational leaves like yellow and brown color leaves. This is reflected in the lower DC of barley side- and top-view test images (0.9384 and 0.9617) compared to the arabidopsis top-view and maize models (> 0.97). Also, the trained model exhibited a low value of DC (0.9117) for the arabidopsis side view test image compared to the other models due to the low contrast of secondary stems which have intensity similar to the background pixels.

In addition, the trained arabidopsis top-view model is validated on reference data sets including examples of stressed and multi-tray experiments. Our experimental results showed that the model achieved a remarkably high DC of 0.9664 (stressed plants) and 0.9873 (multi-tray images) on these unseen data. However, small background noisy objects which have intensity and patterns similar to the leaves require additional application morphological operations (e.g., min cluster size) that are also available with the DeepShoot GUI tool. Furthermore, the model achieved a very high DC (> 0.9), especially on untrained images with a different background from Scharr et al. dataset. Overall our results indicate that the CNN model trained a particular set of images can also be applied to unseen data exhibiting similar plant shoot patterns but different background regions.

The performance of the proposed U-net was compared with the shallow learning neural networks. Thereby, it was shown that most of the arabidopsis and maize images have a relatively low discrepancy between the predicted DC of both algorithms, because these images contain, mostly, high contrast green color pixels for the target structures. In contrast, the shallow neural network exhibited a significantly lower DC on barley images. We draw the observation back to the fact that barley plants have more variable color fingerprints including brown and yellow leaves. This shows that the neural network is only capable of segmenting high contrast shoot structures, whereas the U-net model is capable of segmenting both high contrast and color-altering shoot structures. Because CNN frameworks are capable of generating multi-level features including neighborhood information, color, spatial patterns, and textural features compared to shallow learning methods where only neighborhood information was calculated. Therefore, rich information makes DeepShoot outperforms shallow networks. Furthermore, tests of computational performance of the shallow neural network vs. the proposed U-net model demonstrated the superior performance of the latter. In summary, the DeepShoot tool enables users to perform segmentation and analysis of plant shoot images faster and more accurately in comparison to the shallow neural network.

Furthermore, the performance of the proposed U-net model is compared with vgg19, resnet50, and xception encoder backbones. Thereby, it was observed that lower depth architecture vgg19 achieved better results in comparison to deep depth architectures such as resnet50 and xception that tend to overfit. This can be attributed to the higher complexity of these multi-layer networks that generate too many redundant features. However, the vgg19 model still contains a large number of convolution layers with trainable weights which makes it 10 times larger in size than our proposed U-net. Therefore, our proposed model achieves optimum results at the lower level of complexity which enables us to perform high-throughput plant phenotyping on both lower and higher hardware configuration systems in real time.

It is known that U-net captures not only color but also spatial pattern information. From this perspective, one can expect larger segmentation errors by application of DeepShoot to optical scenes strongly deviating from plant and background structures used by our model training. Nevertheless, our tests with unseen shoot images indicated that the present CNN framework can also be applied to the analysis of quite different optical scenes or filed-like images as long as the target plant structures are optically somewhat similar to images used in our training sets. Users are free to try and evaluate the performance of provided segmentation models on their particular images. From that perspective, there are no other restrictions as the requirement of RGB image with the size ≥256 x 256.

Moreover, segmentation of thin or twisted leaves, flowers as well as shadowed or light-reflecting regions (such as metallic surfaces) is more prone to misclassification, which in turn may lead to fracturing of targeted structures or false-segmented background regions. Nevertheless, improvements in model accuracy and generability can be certainly expected by extending the training set of ground truth images with more and more variable data, in particular, more examples of stressed/aged phenotypes exhibiting non-green colors, e.g., brown, yellow, red leaves. Furthermore, the tool can be extended by automated detection of the plant type and the camera view (side or top) that have to be manually selected in the present implementation from the list of pre-trained CNN models. Finally, further investigations are required to quantitatively assess and compare different model architectures as well as the performance of binary vs. multi-class segmentation models.

## Data Availability Statement

The raw data supporting the conclusions of this article will be made available by the authors, without undue reservation.

## Author Contributions

NN, MH, and EG conceived, designed, and performed the computational experiments, analyzed the data, wrote the manuscript, prepared figures, and tables, and reviewed drafts of the manuscript. KN executed the laboratory experiments, acquired image data, and reviewed drafts of the manuscript. FS read and corrected the drafts of the manuscript. TA co-conceptualized the study and reviewed the manuscript. All authors contributed to the article and approved the manuscript in its present form.

## Funding

This study was funded by Deutsche Forschungsgemeinschaft (DFG, German Research Foundation) - HE 9114/1-1. MH was supported from European Regional Development Fund-Project SINGING PLANT (No. CZ.02.1.01/0.0/0.0/16 026/0008446).

## Conflict of Interest

The authors declare that the research was conducted in the absence of any commercial or financial relationships that could be construed as a potential conflict of interest.

## Publisher's Note

All claims expressed in this article are solely those of the authors and do not necessarily represent those of their affiliated organizations, or those of the publisher, the editors and the reviewers. Any product that may be evaluated in this article, or claim that may be made by its manufacturer, is not guaranteed or endorsed by the publisher.
