# Supplementary material for: Deep Learning Based Greenhouse Image Segmentation and Shoot Phenotyping (DeepShoot)
Source: Front Plant Sci. 2022 Jul 13;13:906410. doi: 10.3389/fpls.2022.906410 (PMC9328757; doi:10.3389/fpls.2022.906410)
Supplement: Supplementary file 1 [file Data_Sheet_1.PDF]

Supplementary Material:

Deep learning based greenhouse image segmentation and shoot phenotyping (DeepShoot),

N. Narisetti*, M. Henke, K. Neumann, T. Altmann, E. Gladilin*.

Table S1. Shoot Traits: The description of estimated shoot phenotypic traits in DeepShoot software.

Feature name	Description
Shoot Area	Total number of pixels in the segmented shoot image
BBX Area	Total number of pixels in the bounding box of the segmented shoot image
Shoot Area to BBX Area	Ratio of actual shoot area to bounding box area
BBX Height	Bounding box height of the segmented shoot image
BBX Width	Bounding box width of the segmented shoot image
Mean X	Mean of the geometrical distribution of segmented shoot pixels in horizontal direction
Stdev X	Standard deviation of the geometrical distribution of segmented shoot pixels in horizontal direction
Mean Y	Mean of the geometrical distribution of segmented shoot pixels in vertical direction
Stdev Y	Standard deviation of the geometrical distribution of segmented shoot pixels in vertical direction
CH Area to BBX Area	Ratio of convex-hull area to the bonding box area of segmented shoot image
Shoot Area to CH Area	Ratio of actual number of segmented pixels to convex-hull area
R mean, G mean, B mean	Mean value of red, green and blue (RGB) channels of segmented shoot image respectively
R stdev, G stdev, B stdev	Standard deviation of red, green and blue (RGB) channels of segmented shoot image respectively
H mean, S mean, V mean	Mean value of hue, saturation and value (HSV) channels of segmented shoot image respectively
H stdev, S stdev, V stdev	Standard deviation of hue, saturation and value (HSV) channels of segmented shoot image respectively
L mean, a mean, b mean	Mean value of CIE Lab channels of segmented shoot image respectively
L stdev, a stdev, b stdev	Standard deviation of CIE Lab channels of segmented shoot image respectively
R mean, G mean, B mean	Mean value of red, green and blue (RGB) channel of segmented shoot image respectively
R stdev, G stdev, B stdev	Standard deviation of red, green and blue (RGB) channel of segmented shoot image respectively
hny_H mean, hny_S mean, hny_Y mean	Mean value of hue, saturation and luminance (HSY) channel of segmented shoot image respectively
hny_H stdev, hny_S stdev, hny_Y stdev	Standard deviation of hue, saturation and luminance (HSY) channel of segmented shoot image respectively

Figure S1: Impact of learning rate (lr) on model training. X-axis represents number of iteration model trained and y-axis represents calculated binary cross entropy loss.

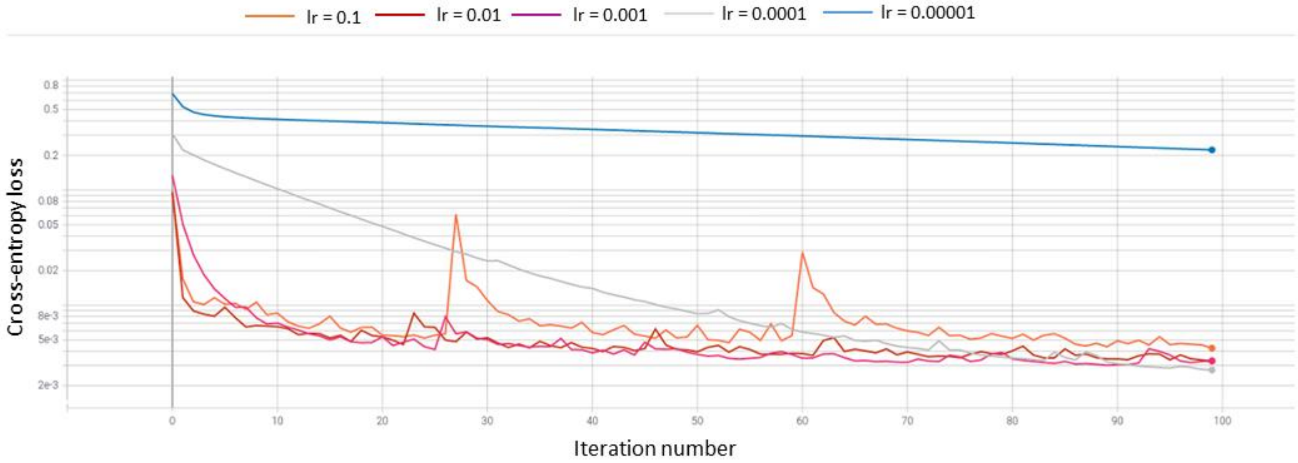

Note: Model with learning rate 0.001 represents optimally trained model with exponential reduction in loss at initial iterations and stabilized loss over the iterations.
